# School related factors and 1yr change in physical activity amongst 9–11 year old English schoolchildren

**DOI:** 10.1186/1479-5868-9-153

**Published:** 2012-12-31

**Authors:** Joyce A Mantjes, Andrew P Jones, Kirsten Corder, Natalia R Jones, Flo Harrison, Simon J Griffin, Esther MF van Sluijs

**Affiliations:** 1 Center for Human Movement Sciences, University Medical Center Groningen, University of Groningen, Section F, PO Box 196, Groningen, 9700 AD, The Netherlands; 2 School of Environmental Sciences, University of East Anglia, Norwich, NR4 7JT, UK; 3 Medical Research Council Epidemiology Unit, Institute of Metabolic Science, Box 285, Addenbrookes Hospital, Hills Road, Cambridge, CB2 0QQ, UK; 4 UK CRC Centre for Diet and Activity Research, Institute of Public Health, Robinson Way, Cambridge, CB2 0SR, UK

**Keywords:** Physical activity, School, Change, Children, Determinants

## Abstract

**Background:**

Activity levels are known to decline with age and there is growing evidence of associations between the school environment and physical activity. In this study we investigated how objectively measured one-year changes in physical activity may be associated with school-related factors in 9- to 10-year-old British children.

**Methods:**

Data were analysed from 839 children attending 89 schools in the SPEEDY (Sport, Physical Activity, and Eating behaviours: Environmental Determinants in Young People) study. Outcomes variables were one year changes in objectively measured sedentary, moderate, and vigorous physical activity, with baseline measures taken when the children were 9–10 years old. School characteristics hypothesised to be associated with change in physical activity were identified from questionnaires, grounds audits, and computer mapping. Associations were examined using simple and multivariable multilevel regression models for both school (9 am – 3 pm) and travel (8–9 am and 3–4 pm) time.

**Results:**

Significant associations during school time included the length of the morning break which was found to be supportive of moderate (β coefficient: 0.68 [p: 0.003]) and vigorous (β coefficient: 0.52 [p: 0.002]) activities and helps to prevent adverse changes in sedentary time (β coefficient: -2.52 [p: 0.001]). During travel time, positive associations were found between the presence of safe places to cross roads around the school and changes in moderate (β coefficient: 0.83 [p:0.022]) and vigorous (β coefficient: 0.56 [p:0.001]) activity, as well as sedentary time (β coefficient: -1.61 [p:0.005]).

**Conclusion:**

This study suggests that having longer morning school breaks and providing road safety features such as cycling infrastructure, a crossing guard, and safe places for children to cross the road may have a role to play in supporting the maintenance of moderate and vigorous activity behaviours, and preventing the development of sedentary behaviours in children.

## Introduction

Despite the well-known health benefits of physical activity in children [1-3], activity levels are known to decline with age [4-8]. In the SPEEDY (Sport, Physical Activity, and Eating behaviours: Environmental Determinants in Young People) study, a longitudinal study of 9 to 10 year old British children which also forms the basis for the present analyses, a significant decrease in physical activity outside school was observed over a period as short as one year [9]. To prevent such declines, it is important to identify potential causal factors that may be driving them. Social-ecological models of health suggest that the physical and policy environment contexts are likely to be an important driver of general health related behaviours [10] and physical activity specifically [11]. Given that children spend much of their time at school during significant parts of the year, social-ecological models posit that factors relating to the physical and policy environments of schools may be important determinants of their physical activity levels [12].

In two recent studies, features of the school environment have been significantly related to accelerometer counts in adolescent girls [13] and in primary school students [14]. Moreover, variation in physical activity between schools has been reported, with a distinction being apparent between those offering interschool physical activity programs [14]. Furthermore, cross-sectional positive associations have previously been observed between moderate and vigorous activity and the provision of school sports facilities and active travel infrastructure in the sample used for the present analyses (SPEEDY) [15].

Whilst the current evidence suggests that the school may be an important influence on activity levels, the cross-sectional nature of many studies is a limitation. Previous studies found a decline of physical activity as children get older, continuing into adolescence and adulthood [9,16,17]. However it is not known what factors are associated with this decline and hence how to prevent it. Few studies have been undertaken that attempt to identify potential environmental determinants of changes of physical activity in children, and none have had a specific focus on the role of the school [18]. This is despite the fact that there is some evidence of the potential of school based interventions to increase physical activity in children and adolescents [19]. Successful interventions have included the redesign of school grounds, and the provision of additional play equipment and lessons to promote healthy behaviours [19-21]. These studies highlight the potentially important role schools may play in changing physical activity, as well as their untested potential in preventing declines in the first place.

With the growing evidence for associations between the school environment and physical activity, it is timely to consider how observed changes in physical activity in child cohorts may be associated with school-related factors. Therefore, the aim of this study was to build upon our previous cross-sectional work examining the school-level correlates of physical activity in SPEEDY [15] by investigating the relationship between school policy and environment-related factors, and 1 year changes in objectively measured school time physical activity in 9- to 10-year-old British children.

## Methods

### Study design & sample

SPEEDY was set up to identify determinants of physical activity and dietary habits in 9–10 year old (school Year 5) British school children in the county of Norfolk, United Kingdom. Ethical approval for the study was obtained from the University of East Anglia research ethics committee. A detailed overview of the study methods and sampling are described elsewhere [22]. Briefly, after purposively sampling schools to achieve urban–rural heterogeneity, 92 schools were recruited from 157 initially approached. Each school was visited and information packs were distributed to 3619 eligible children, resulting in 2064 children (57% of the eligible sample) participating in baseline measurement between April and July 2007. Follow-up was undertaken between April and July 2008, whereby information sheets and consent forms were mailed to all 2064 children. An accelerometer and instruction sheets were mailed to those who consented to participate in follow-up.

### Physical activity

Physical activity was measured at both baseline and follow-up using an Actigraph accelerometer (model GT1M [Actipraph, Pensacola, FL]). At baseline the Actigraphs were fitted on the children at school, whilst at follow-up they were sent to the children by post. Children received wearing instructions and were asked to wear the monitors during waking hours for 7 days except when bathing, showering and swimming. The monitors were set to store activity data at 5-second intervals. A bespoke programme (MAHUffe [Medical Research Council Epidemiology Unit, Cambridge, United Kingdom]) was used for processing activity data. The outcome variables recorded were minutes of sedentary time (≤100 counts/minute), moderate activity (2000–3999 counts/minute), and vigorous activity (>4000 counts/min) [23,24] on weekdays between 8am and 4pm (school and travel time). Periods of ≥ 10 minutes of zero activity were classed as non-wear time. Days with <500 minutes of recording, and participants with <3 days out of 7 of recording for baseline, follow-up, or both were excluded. These exclusion criteria have been previously found to improve the reliability of estimates of children’s physical activity [25].

### Biological & demographic factors

At baseline, children self-reported their date of birth and age was subsequently calculated using the measurement date. Also at baseline, child height and weight was measured by trained researchers visiting each school. Weight was measured to the nearest 0.1 kilogram using a non-segmental bio impedance scale (Tanita type TBF-300A). Portable Leicester height measures were used to measure height to the nearest millimetre. From this, Body Mass Index (BMI) was calculated. In terms of demographic covariates, parents self-reported level of education and reported ethnicity were obtained from a questionnaire survey.

### School environment

In order to inform sampling, general school information was provided by Norfolk County Council. This included whether schools participated in the Healthy Norfolk Schools (health education) programme, as well as the number of pupils in the study’s target year group (the number of pupils in Year 4 during the 2005–2006 academic year who would be in Year 5 during data collection). Based on the school postcode (zipcode), an urban/rural classification was determined using the four population density profiles of Bibby and Shepherd’s classification of rurality [26].

Several methods were used to obtain information about the schools regarding policies, facilities, grounds and the neighbourhood. A questionnaire was completed at baseline by the head teacher of each school which requested information on physical activity opportunities at school, school rules and attitudes, and certain features of the physical environment around the school such as the availability of changing facilities, sports equipment and the presence of heavy traffic. More information on the measures used in this questionnaire is available elsewhere [15]. To measure the suitability of the school grounds for physical activity, a validated grounds audit (completed by five trained auditors) was completed at baseline (see Jones et al. for details on development, validity and reliability [27]). The audit contained 44 items and provided six composite scores which described different elements of environmental supportiveness for physical activity. A higher value on each score indicated greater supportiveness. The six composite scores were: walking provision (e.g. existence of pavements/sidewalks), cycling provision (e.g. existence of cycle parking facilities), sports and play provision (e.g. number of sports pitches), other non-activity facility provision (e.g. number of benches for seating), design of the school grounds (e.g. if the school is on a split-site), and aesthetics (e.g. presence of trees). Reliability testing showed that all except one of the components had mean inter-rater percentage agreements of 90% or above, with just walking provision being lower at 76%, but still indicating better than fair agreement [27].

At baseline, objective measures of the supportiveness of the neighbourhood around the school for physical activity were computed using ArcGIS 9.2 Geographic Information System (GIS). For these indicators, the neighbourhood was defined as the area within 800m (roughly equating to a 10 minute walk) along the pedestrian network (streets and footpaths) from the school boundary. The variables computed have been published previously [28] and included measures of the presence of physical activity facilities, accessible open land, road traffic accidents, the presence of major roads, density of verges, effective walkable area (defined as the ratio of the area within an 800m walking distance along the pedestrian network from the school to the area within an 800m radius of the school), connected node ratio (the ratio of junctions to junctions and cul-de-sacs; an indicator of the connectivity of the street network), and the Herfindahl-Hirschman index (a measure of the diversity of land uses in the school neighbourhood). These measures have been commonly employed to measure environmental supportiveness in other studies and were computed according to standard procedures [29].

### Statistical analysis

Associations between school characteristics and changes in the percentage of time spent in sedentary, moderate, and vigorous activity between baseline and follow-up were examined using regression models. Both baseline and follow-up values were calculated as a percentage of accelerometer wear time to adjust for different wear times at baseline and follow-up. Change in physical activity was then calculated as the difference between these follow-up and baseline percentages (i.e. percentage sedentary activity follow-up – percentage sedentary activity baseline) using the methodology of Corder et al. [9]. Changes in percentages were fitted as the dependent variables in all models, with the baseline percentage value being modelled as a covariate.

Separate analyses were undertaken for each activity intensity and different times of the day. School time was defined as the period between 9 am and 3 pm, whilst travel time was the combined periods between 8 am – 9 am and 3 pm – 4 pm. Relevant explanatory variables were selected for each of these two time periods based on the theoretical relevance of the items considered (i.e. policy regarding travelling was only examined for travel time physical activity). As children were sampled within schools, 2-level multilevel models were fitted using the ‘xtmixed’ command in Stata 11.0 (Statacorp Inc), with child at level 1 and school at level 2.

To fit the regression models, partially adjusted associations were examined for each school variable, where adjustment was made for sex, age, BMI, parental education, ethnicity and baseline physical activity. As a large number of variables were tested, rather than enter them all into a multivariable model, separate multivariable multilevel models were initially constructed for all variables grouped under the categories of school characteristics (6 factors), school policy (17 factors), school facilities (11 factors), school grounds (7 factors), and school neighbourhood (15 factors). From each model, non-significant variables were removed by manual backward elimination based on p<0.1. Finally, remaining variables in these four models were added to a fully adjusted model and again backwards elimination was used until only items that were statistically significant at p<0.05 remained. For all associations, a positive regression coefficient indicates less of a decrease or more of an increase in physical activity, whilst a negative association means more of a decrease or less of an increase in physical activity. The amount of residual variation in the outcomes that was associated with the school each child attended was calculated in the form of intraclass correlation coefficients (ICCs).

## Results

Of the 2064 children providing baseline measures, 1019 (49.4%) provided some follow-up measurements, of which 954 (93.6%) returned an activity monitor containing data. After exclusion of invalid accelerometry, a total of 839 (40.6% of the original sample) children in 89 (96.7% of original sample) different schools remained for analysis.

Table 1 shows the characteristics of these participants, and their activity levels. At baseline around 70% of school time was recorded as being sedentary according to the accelerometry, and during the commuting period this was 55%. The percentage of time spent in moderate and vigorous activity was generally low, but was higher during travel times compared to the school day. Changes between baseline and follow-up in activity across the different intensities were generally small and positive. In girls only, some declines in vigorous activity were observed in both time periods and in moderate activity during the travel period.

**Table 1:** Baseline characteristics of the SPEEDY study sample for participants with valid physical activity (PA) data (N=839), baseline PA values (% of worn time) and changes in PA % for school time and travel time

		**Proportion/mean (SD)**		
**Measures**	**Activity intensity**	**Total (N = 839)**	**Boys (N = 349)**	**Girls (N = 490)**
Age (years)		10.23 (0.30)	10.22 (0.29)	10.24 (0.31)
BMI (kg/m^2^)		18.00 (3.04)	17.74 (2.74)	18.18 (3.23)
Parental education: a-levels or higher		41.8%	45.0%	39.6%
White ethnicity		97.0%	97.4%	96.7%
***School time (9-15h)***				
Baseline PA (% of worn time)	Sedentary	69.49 (5.82)	66.99 (5.99)	71.27 (4.98)
	Moderate	5.23 (1.58)	6.09 (1.62)	4.62 (1.23)
	Vigorous	2.96 (1.53)	3.67 (1.70)	2.45 (1.16)
Change PA (change in %)	Sedentary	−0.19 (6.08)	−0.75 (6.42)^+^	0.20 (5.80)
	Moderate	0.18 (1.75)^++^	0.27 (1.88)^++^	0.11 (1.65)
	Vigorous	−0.04 (1.50)	0.10 (1.79)	−0.13 (1.25)^+^
***Travel time (8-9h, 15-16h)***				
Baseline PA (% of worn time)	Sedentary	55.92 (8.12)	54.33 (8.65)	57.06 (7.52)
	Moderate	10.52 (4.78)	10.82 (4.72)	10.31 (4.81)
	Vigorous	3.41 (2.55)	4.07 (2.81)	2.95 (2.24)
Change PA (change in %)	Sedentary	0.40 (8.30)	−0.30 (8.61)	0.90 (8.04)^+^
	Moderate	0.07 (4.69)	0.29 (4.39)	−0.08 (4.89)
	Vigorous	0.07 (4.69)	0.33 (2.67)^+^	−0.12 (2.23)

^+^ p value change in physical activity <0.05; ^++^ p value change in physical activity <0.01.

The partially adjusted regression models for school time are shown in Table 2. Various associations were observed with all three outcomes. Whilst longer morning breaks were supportive of positive changes in moderate and vigorous activity, and less sedentary behaviours, the existence of sports and play equipment and changing facilities was associated in an opposite and hence counter-intuitive direction.

**Table 2:** Associations with school factors in partially adjusted multilevel linear regression models predicting change in percentage of sedentary, moderate and vigorous physical activity during school time (9-15h)

**Exposure variables**	**Percentage prevalence/mean (SD)**	**Sedentary (% change)**	**Moderate (% change)**	**Vigorous (% change)**
		**β Coefficient (95% CI)**	**β Coefficient (95% CI)**	**β Coefficient (95% CI)**
***School characteristics***				
Location town fringe (y/n [reference: urban])^a^	34.4%	0.84 (−0.52 – 2.21)	−0.37 (−0.78 – 0.05)	−0.09 (−0.39 – 0.20)
Location village/hamlet dwelling (y/n [reference: urban])^a^	27.5%	−1.14 (−2.40 – 0.12)	0.27 (−0.12 – 0.65)	0.26 (−0.01 – 0.53)
Number of year 4 children in 2006 ^a^	54.41 (34.29)	0.01 (−0.01 – 0.03)	−0.00 (−0.01 – 0.01)	−0.00 (−0.01 – 0.00)
Participation in healthy school program (y/n)^a^	36.1%	0.94 (−0.33 – 2.21)	−0.17 (−0.56 – 0.23)	−0.12 (−0.41 – 0.16)
Morning break length >15 minutes (y/n)^b^	13.8%	−2.36 (−4.05 - -0.67)^++^	0.84 (0.34 – 1.34)^++^	0.64 (0.28 – 0.99) ^+++^
Lunch break length (minutes)^b^	57.89 (6.68)	−0.10 (−0.19 - -0.02)^+^	0.03 (0.00 – 0.05)	−0.00 (−0.02 – 0.02)
***School policy***				
Breaks: allowed to play outside in bad weather (y/n)^b^	6.7%	−0.40 (−2.89 – 2.08)	0.36 (−0.39 – 1.11)	−0.11 (−0.65 – 0.43)
Breaks: screen play allowed (y/n)^b^	65.3%	0.68 (−0.65 – 2.02)	−0.27 (−0.68 – 0.14)	0.04 (−0.26 – 0.33)
Breaks: ≥ 2 physical activities allowed (y/n)^b^	52.1%	0.01 (−1.23 – 1.26)	0.10 (−0.27 – 0.48)	−0.24 (−0.51 – 0.02)
Provide …physical activity information (y/n)^b^	87.4%	0.76 (−1.27 – 2.80)	−0.09 (−0.72 – 0.53)	−0.06 (−0.50 – 0.38)
…health promotion information (y/n)^b^	78.3%	1.94 (0.44 – 3.44)^+^	−0.39 (−0.86 – 0.07)	−0.33 (−0.67 – -0.00)^+^
…risks of unhealthy lifestyle information (y/n)^b^	72.1%	1.00 (−0.40 – 2.39)	−0.22 (−0.65 – 0.20)	−0.09 (−0.39 – 0.21)
Hours of physical education ^b^	2.08 (0.49)	1.52 (0.35 – 2.69)^+^	−0.28 (−0.64 – 0.08)	−0.10 (−0.36 – 0.17)
Extracurricular PA available lunch breaks (y/n)^b^	70.7%	1.14 (−0.13 – 2.42)	−0.36 (−0.74 – 0.03)	−0.27 (−0.55 – 0.00)
***School facilities***				
Existence of …gym facility (y/n)^b^	58.3%	−0.34 (−1.66 – 0.97)	0.17 (−0.22 – 0.57)	0.28 (−0.00 – 0.56)
…indoor sports facility (y/n)^b^	58.6%	−0.45 (−1.81 – 0.90)	−0.00 (−0.41 – 0.41)	−0.11 (−0.39 – 0.18)
… sports field/pitch facility (y/n)^b^	94.5%	−0.39 (−3.14 – 2.37)	−0.18 (−1.02 – 0.65)	0.20 (−0.40 – 0.79)
… pool facility (y/n)^b^	38.6%	−0.31 (−1.60 – 0.99)	0.17 (−0.23 – 0.56)	0.04 (−0.24 – 0.32)
… changing facilities (y/n)^b^	55.8%	1.24 (0.02 – 2.45)^+^	−0.41 (−0.78 – -0.05)^+^	−0.27 (−0.53 – -0.00)^+^
… play equipment (y/n)^b^	95.1%	3.71 (0.06 – 7.36)^+^	−0.47 (−1.61 – 0.68)	−0.05 (−0.86 – 0.75)
… sports equipment (y/n)^b^	98.7%	9.04 (3.93 – 14.16)^++^	−3.52 (−5.00 – -2.04)^+++^	−2.52 (−3.54 – -1.50)^+++^
Use of local park or playground (y/n)^b^	16.1%	−0.30 (−1.90 – 1.30)	0.13 (−0.35 – 0.62)	0.21 (−0.14 – 0.56)
Medium or high quality of sports facilities (y/n)^b^	75.7%	1.01 (−0.39 – 2.42)	−0.46 (−0.90 – -0.01)^+^	−0.29 (−0.59 – 0.01)
Physical activity facility score (max: 17)^c^	8.30 (1.91)	0.06 (−0.28 – 0.39)	−0.03 (−0.14 – 0.07)	−0.05 (−0.12 – 0.02)
Other facility score (max: 12)^c^	3.49 (1.59)	−0.08 (−0.48 – 0.32)	0.02 (−0.10 – 0.15)	−0.04 (−0.13 – 0.04)
***School grounds***				
Playground area (km^2^)^b^	11.82 (7.38)	0.07 (−0.01 – 0.15)	−0.02 (−0.04 – 0.00)	−0.01 (−0.03 – 0.01)
School ground score (min: 1, max: 10)^c^	9.14 (0.80)	−0.12 (−0.92 – 0.68)	0.02 (−0.21 – 0.26)	−0.05 (−0.22 – 0.13)
Aesthetics score (min: 3, max: 28)^c^	21.54 (2.24)	−0.11 (−0.39 – 0.16)	0.02 (−0.06 – 0.11)	0.03 (−0.03 – 0.09)

^+^ p<0.05; ^++^ p<0.01; ^+++^ p<0.001.

All models were adjusted for sex, age, BMI, parental education, ethnicity and the baseline value of physical activity.

^a^ Factors derived by the Norfolk County Council (%=yes); ^b^ Factors derived by the school questionnaire (%=yes); ^c^ Factors derived by the audit tool; minimum score = 0 unless stated otherwise.

Table 3 shows the partially adjusted models for travel times. More statistically significant associations were present than for the school day. Living in a village or isolated settlement appeared less supportive of the maintenance of PA, whilst the presence of greater number of same age children was supportive. There were several characteristics of the school policy and physical environment that were also supportive including the presence of a lollypop person (crossing guard) and cycling infrastructure, the existence of safe places to cross the road, fewer road traffic accidents in the school neighbourhood, and a higher land use diversity around the school (measured by lower scores in the Herfindal-hirschman index).

**Table 3:** Associations with school factors in partially adjusted multilevel linear regression models predicting changes in percentage of sedentary, moderate and vigorous physical activity (PA) during travel time (8–9, 15-16h)

**Exposure variables**	**Percentage prevalence/mean (SD)**	**Sedentary (% change)**	**Moderate (% change)**	**Vigorous (% change)**
		**β Coefficient (95% CI)**	**β Coefficient (95% CI)**	**β Coefficient (95% CI)**
***School characteristics***				
Location town fringe (y/n [reference: urban])^a^	34.4%	−1.05 (−2.34 – 0.23)	0.59 (−0.23 – 1.41)	0.10 (−0.27 – 0.47)
Location village/hamlet dwelling (y/n [reference: urban])^a^	27.5%	1.84 (0.62 – 3.07)^++^	−1.62 (−2.39 - -0.85)^+++^	−0.50 (−0.86 - -0.15)^++^
Number of year 4 children in 2006 ^a^	54.41 (34.29)	−0.04 (−0.05 – -0.02)^+++^	0.03 (0.02 – 0.04)^+++^	0.01 (0.00 – 0.01)^++^
Participation in healthy school program (y/n)^a^	36.1%	0.11 (−1.17 – 1.39)	0.05 (−0.76 – 0.86)	0.11 (−0.24 – 0.47)
***School policy***				
Existence of …travel plan (y/n)^b^	87.1%	0.50 (−1.44 – 2.45)	0.31 (−0.90 – 1.53)	0.49 (−0.06 – 1.04)
…walking bus (y/n)^b^	1.8%	0.60 (−3.65 – 4.84)	0.06 (−2.57 – 2.70)	−0.41 (−1.62– 0.79)
…park and stride (y/n)^b^	16.1%	0.54 (−1.06 – 2.14)	−0.52 (−1.52 – 0.48)	−0.11 (−0.57 – 0.34)
… breakfast club (y/n)^b^	35.8%	−0.33 (−1.59 – 0.93)	0.36 (−0.44 – 1.15)	0.18 (−0.17 – 0.54)
… lollypop person (y/n)^b^	49.3%	−1.74 (−2.91 – -0.57)^++^	1.40 (0.67 – 2.12)^+++^	0.37 (0.03 – 0.70)^+^
Provide …physical activity information (y/n)^b^	87.4%	−0.36 (−2.29 – 1.56)	0.30 (−0.91 – 1.51)	0.06 (−0.48 – 0.60)
…health promotion information (y/n)^b^	78.3%	0.83 (−0.65 – 2.32)	−0.13 (−1.08 – 0.82)	0.16 (−0.27 – 0.58)
…risks of unhealthy lifestyle information (y/n)^b^	72.1%	−0.39 (−1.77 – 0.98)	0.33 (−0.53 – 1.19)	0.15 (−0.24 – 0.53)
… cycle training (y/n)^b^	93.7%	−0.89 (−3.27 – 1.48)	0.95 (−0.52 – 2.41)	0.04 (−0.63 – 0.70)
… pedestrian training (y/n)^b^	34.4%	1.34 (0.15 – 2.54)^+^	−1.28 (−2.02 – -0.54)^++^	−0.21 (−0.56 – 0.14)
Extracurricular PA available before school (y/n)^b^	10.4%	−0.37 (−2.46 – 1.72)	0.66 (−0.65 – 1.97)	0.25 (−0.33 – 0.82)
Extracurricular PA available weekends (y/n)^b^	33.8%	−1.77 (−3.03 – -0.51)^++^	1.14 (0.34 – 1.94)^++^	0.21 (−0.16 – 0.58)
***School grounds***				
Existence of a bike rack (y/n)^b^	88.3%	−1.12 (−2.99 – 0.74)	0.97 (−0.19 – 2.13)	−0.05 (−0.58 – 0.48)
Existence of an entrance for pedestrians/cyclists only (y/n)^b^	70.0%	0.27 (−1.08 – 1.61)	−0.11 (−0.94 – 0.72)	0.02 (−0.36 – 0.41)
Playground area (km^2^)^b^	11.82 (7.38)	−0.08 (−0.16 – 0.00)	0.05 (−0.00 – 0.10)	0.01 (−0.02 – 0.03)
School ground score (min: 1, max: 10)^c^	9.14 (0.80)	−0.18 (−0.96 – 0.60)	0.08 (−0.41 – 0.57)	−0.10 (−0.32 – 0.13)
Walking access score (max: 5)^c^	2.24 (0.74)	−0.49 (−1.30 – 0.33)	0.44 (−0.07 – 0.96)	0.26 (0.04 – 0.48)^+^
Cycling access score (max: 9)^c^	3.70 (1.25)	−0.60 (−1.06 – -0.14)^+^	0.48 (0.19 – 0.76)^++^	0.11 (−0.02 – 0.25)
Aesthetics score (min: 3, max: 28)^c^	21.54 (2.24)	0.14 (−0.13 – 0.41)	−0.12 (−0.29 – 0.05)	−0.02 (−0.10 – 0.06)
***School neighbourhood***				
Existence of …heavy traffic (y/n)^b^	27.2%	−1.19 (−2.40 – 0.02)	0.76 (−0.00 – 1.52)	0.26 (−0.09 – 0.60)
…pathways near school (y/n)^b^	84.4%	−1.06 (−2.65 – 0.54)	0.89 (−0.11 – 1.89)	−0.04 (−0.50 – 0.42)
…safe places to cross roads (y/n)^b^	34.6%	−1.76 (−3.01 – -0.52)^++^	1.33 (0.53 – 2.12)^++^	0.66 (0.32 – 1.00)^+++^
Cars drive slowly (y/n)^b^	18.6%	1.48 (−0.01 – 2.98)	−0.70 (−1.64 – 0.24)	0.07 (−0.37 – 0.50)
Streets are safe to walk or ride (y/n)^b^	30.4%	0.58 (−0.73 – 1.89)	−0.37 (−1.20 – 0.45)	0.11 (−0.27 – 0.48)
Streets are free from rubbish (y/n)^b^	69.8%	0.88 (−0.46 – 2.22)	−0.53 (−1.39 – 0.32)	−0.26 (−0.63 – 0.12)
Easy to get to school by foot (y/n)^b^	77.5%	−1.30 (−2.70 – 0.10)	0.70 (−0.20 – 1.59)	0.20 (−0.20 – 0.60)
Number of PA facilities per km^2 d^	2.69 (2.84)	−0.19 (−0.39 – 0.02)	0.17 (0.04 – 0.30)^++^	0.05 (−0.01 – 0.11)
Percentage of accessible land ^d^	1.90 (5.33)	0.00 (−0.10 – 0.11)	−0.01 (−0.07 – 0.06)	0.00 (−0.03 – 0.03)
Number of traffic accidents per km of road ^d^	1.88 (1.76)	−0.30 (−0.63 – 0.02)	0.31 (0.10 – 0.51)^++^	0.11 (0.02 – 0.20)^+^
Proportion of roads that are A roads ^d^	0.06 (0.08)	−6.27 (−13.63 – 1.08)	3.02 (−1.69 – 7.73)	1.44 (−0.63 – 3.51)
m^2^ verge per m of road ^d^	1.85 (0.93)	0.31 (−0.31 – 0.94)	−0.42 (−0.81 – -0.03)^+^	−0.19 (−0.36 – -0.02)^+^
Effective walkable area ratio ^d^	0.52 (0.13)	3.67 (−1.01 – 8.35)	−2.30 (−5.25 – 0.66)	−0.41 (−1.74 – 0.92)
Connected node ratio ^d^	0.71 (0.09)	7.83 (1.49 – 14.18)^+^	−4.23 (−8.32 – -0.13)^+^	−0.01 (−1.88 – 1.86)
Herfindahl-hirschman index ^d^	2.72 (1.08)	0.56 (0.04 – 1.08)^+^	−0.38 (−0.70 – -0.05)^+^	−0.20 (−0.35 – -0.06)^++^

^+^ p<0.05; ^++^ p<0.01; ^+++^ p<0.001.

All models were adjusted for sex, age, BMI, parental education, ethnicity and the baseline value of physical activity.

^a^ Factors derived by the Norfolk County Council (%=yes); ^b^ Factors derived by the school questionnaire (%=yes); ^c^ Factors derived by the audit tool; minimum score = 0 unless stated otherwise; ^d^ Factors derived by GIS.

In the final fully adjusted models (Table 4), a number of associations observed during the school day were in a counter-intuitive direction, including the provision of health promotion information (sedentary: β=1.93, CI=0.72-3.15, p=0.002), more hours of physical education (PE) (sedentary: β=1.27, CI=0.23-2.31, p=0.016), and the provision of play (sedentary: β=3.29, CI=0.51-6.07, p=0.020) and sports (for all intensities) equipment as well as changing facilities (moderate: β=−0.33, CI=−0.64--0.02, p=0.035). Associations with break length remained in the expected directions for all intensities. When change in physical activity during travel times was considered, a number of associations observed before full adjustment remained, with the existence of safe places to cross the road, and the number of same-age children in the school both being consistently associated in the direction expected with activity at all intensities. In these models, the ICC values for changes in school time physical activity were statistically significant (Sedentary: 0.09; Moderate: 0.135; Vigorous 0.071), but those for changes in travel time physical activity were not (all below 0.02).

**Table 4:** Results from fully-adjusted multivariable multilevel linear regressions for changes in sedentary, moderate and vigorous physical activity (PA), during school time (9-15h) and travel time (8–9, 15-16h)*

**Time period**	**Predictors of activity intensity change**	**β Coefficient**	**95% CI**	**p-value**	**ICC**
School time (9-15h)	***Sedentary time change***				0.090^+++^
	Constant	18.95	3.76 – 34.13	0.014	
	Morning break length >15 minutes (y/n)^b^	−2.52	−4.00 - -1.04	0.001	
	Lunch break length (minutes)^b^	−0.08	−0.16 - -0.00	0.037	
	Provide health promotion information (y/n)^b^	1.93	0.72 – 3.15	0.002	
	Hours of physical education ^b^	1.27	0.23 – 2.31	0.016	
	Existence of play equipment (y/n)^b^	3.29	0.51 – 6.07	0.020	
	Existence of sports equipment (y/n)^b^	7.15	2.81 – 11.49	0.001	
	***Moderate activity change***				0.135^+++^
	Constant	5.38	1.49 – 9.27	0.007	
	Morning break length >15 minutes (y/n)^b^	0.68	0.23 – 1.13	0.003	
	Lunch break length (minutes) ^b^	0.03	0.00 – 0.05	0.018	
	Existence of changing facilities (y/n)^b^	−0.33	−0.64 - -0.02	0.035	
	Existence of sports equipment (y/n)^b^	−2.71	−4.10 - -1.33	<0.001	
	***Vigorous activity change***				0.071^+++^
	Constant	7.62	4.48 – 10.77	<0.001	
	Morning break length >15 minutes (y/n)^b^	0.52	0.19 – 0.85	0.002	
	Provide health promotion information (y/n)^b^	−0.38	−0.66 - -0.11	0.006	
	Existence of sports equipment (y/n)^b^	−2.15	−3.13 - -1.17	<0.001	
Travel time (8–9, 15-16h)	***Sedentary time change***				0.002
	Constant	37.10	19.42 – 54.79	<0.001	
	Number of year 4 children in 2006 ^a^	−0.02	−0.03 - -0.00	0.030	
	Provide physical activity information (y/n)^b^	−2.02	−3.81 - -0.22	0.027	
	Provide health promotion information (y/n)^b^	2.15	0.61 – 3.68	0.006	
	Extracurricular PA available weekends (y/n)^b^	−1.35	−2.47 - -0.23	0.018	
	Existence of safe places to cross roads (y/n)^b^	−1.61	−2.73 - -0.49	0.005	
	***Moderate activity change***				
	Constant	−2.76	−13.20 – 7.67	0.604	<0.001
	Number of year 4 children in 2006 ^a^	0.02	0.01 – 0.03	<0.001	
	Provide physical activity information (y/n)^b^	1.15	0.10 – 2.21	0.033	
	Provide health promotion information (y/n)^b^	−0.93	−1.84 - -0.03	0.043	
	Extracurricular PA available weekends (y/n)^b^	0.68	0.02 – 1.34	0.044	
	Existence of a lollypop person (y/n)^b^	0.68	0.04 – 1.33	0.039	
	Existence of safe places to cross roads (y/n)^b^	0.83	0.12 – 1.54	0.022	
	***Vigorous activity change***				0.016
	Constant	5.63	0.72 – 10.54	0.025	
	Number of year 4 children in 2006 ^a^	0.01	0.00 – 0.01	0.044	
	Existence of safe places to cross roads (y/n)^b^	0.56	0.22 – 0.90	0.001	

^*^All models were adjusted for sex, age, BMI, parental education, ethnicity and the baseline value of physical activity.

^a^ Factors derived by the Norfolk County Council;^ b^ Factors derived by the school questionnaire;^ c^ Factors derived by the audit tool; minimum score = 0 unless stated otherwise;^ d^ Factors derived by GIS, ^+++^ Significant at 0.001 level.

## Discussion

Physical activity is known to decrease as children age and with the growing evidence for associations between the school environment and physical activity, the aim of this study was to examine the relationship between school-related factors and changes in physical activity during both school and travel time. As reported previously, only limited changes in activity were observed during time spent at school and the travel period [9]. However, although the effects were not large, some of the changes that we observed were associated with characteristics of the environment of the school children attended. Overall, more associations were found for sedentary and moderate activity behaviours compared to vigorous physical activity.

During the school day there was evidence that the length of break time may be important in preventing increases in sedentary time and decreases in moderate and vigorous activity, with more favourable outcomes being observed in children attending schools with a break of over 15 minutes. There is evidence that break-time play contributes to overall activity levels in children [30], and thus we suggest that children at schools with longer breaks have the opportunity to play more. Counterintuitive associations were recorded with the existence of changing facilities, and play and sports equipment, with the presence of these facilities being associated with detrimental changes in activity. This might in part be due to the fact that only two schools reported providing no play equipment (32 children) and only one no sports equipment (11 children).

Counter-intuitive negative relationships during the school day were also found with the provision of health promotion information and hours of PE. The association with the provision of information may reflect the fact that the information is more likely to be provided in schools with poorer physical activity outcomes. This is in accordance with previous results of a cross sectional analysis in this sample where a negative association was found between the presence of a physical activity policy and vigorous activity [15]. The association with PE is particularly surprising given that the activity during these lessons should have been recorded by the accelerometry. There is some evidence that the intensity of activity undertaken during PE sessions is surprisingly low [31]. It may be that these compulsory activities do not result in more active children, particularly if children participating in more PE are less active at other times. After adjustment for all the factors in the models, statistically significant between school ICCs remained for the models of change in school-day physical activity. Although their magnitude was small, this suggests that some factors associated with the school were not accounted for in our models. In particular, we did not assess curriculum-based influences, which may be particularly important for time spent sedentary in the classroom.

When changes in travel time physical activity were considered, there was evidence that the number of same age children at the school was supportive of the maintenance of moderate and vigorous activity and helped prevent adverse changes in sedentary behaviours. Schools with more children were more likely to be located in urban areas, and children attending urban schools have previously been shown to be more likely to walk or cycle [32], although the measure of urban–rural status was not significantly associated with change in travel time physical activity in our analysis. One possibility is that more same age children provide better social support for active travel to school, for example by facilitating walking groups or walking with friends. It may also be that some of the physical activity recorded during the travel period was actually from play at either the beginning or end of the journey, and children attending schools with a greater number of friends would be more likely to play. There is evidence that girls attending schools with a greater number of peers have a lower fat mass index [33] and this may be associated with higher levels of active play.

Positive associations with physical activity change during the travel period were also found with two measures of safety (a lollypop person, and reporting of safe crossings) which is in accordance with previous research about associations between physical activity and safety on the route to school [18,34,35]. Whether these provisions are actually promoting active travel or whether the provision of these features is associated with the existing prevalence of active travel to school, requires further investigation.

Although some statistically significant associations were detected, it is noteworthy that many of the variables we tested were not found to be associated with changes in the physical activity outcomes we studied. Although the school audit tool we used had been validated and we measured change in physical activity objectively via accelerometers, it is possible that the lack of associations detected could be due to measurement error with our exposures or outcomes. The fact that many of our exposures showed some associations with each other (for example schools that scored badly on one indicator were more likely to score badly on others) could also explain the lack of associations after adjustment. Alternatively, it could indicate the relative unimportance of the school environment as a determinant of change in physical activity in these intensities. We note that a previous study only found associations with the school environment and overall activity in girls, but not with moderate to vigorous physical activity [13]. This was explained by a possible contribution of the school environment to low intensity activity rather than to activities of moderate to vigorous intensity.

Strengths of this study include the large sample size, and the fact that matched longitudinal data on physical activity and sedentary behaviour change was available for participating children. In addition, change in physical activity was objectively measured using an accelerometer and we made a distinction between behaviours of different intensities. In particular we included sedentary behaviour, which may have an important association with health outcomes [36] but which is often not considered alongside physical activity. A further strength is that we used a wide range of methods (questionnaire, audit, GIS) to characterise components of the school environment with a high degree of detail.

There are however a number of weaknesses. For the analyses, school time was defined as the time between 9 am and 3 pm and travel time as the time between 8 and 9 am and between 3 and 4 pm. However, the precise time at which the school day begins and ends will differ by a small amount between schools (median time schools began: 8.50 am, median time schools ended: 3.15 pm) and some of the activity we recorded during these times may have been at home, in the home neighbourhood, or in the school playground rather than on the journey. A further limitation is that, whilst the accelerometry provides a validated measure of activity intensity, it does not tell us what types of activities the children were engaging in. In addition, we did not have confirmation that the children were still attending the same schools at follow-up measurement compared to the baseline measure, although only 47 of the children taking part in SPEEDY-2 changed address and hence it is unlikely that many attended a different school at follow-up. Our measures of school policy were taken at baseline, and we do not know if they may have changed for the children at follow-up. Some of our exposure measures, in particular the provision of sports and play equipment, varied little between schools, limiting our ability to detect associations. Although limited changes in physical activity levels were observed, sufficient heterogeneity in change was available to study associations. A final limitation is that the large number of tests we undertook means that some of the associations we detected may have been due to chance, although we actually found rather few associations given the large number of variables we tested.

In conclusion, this study found a number of school factors to be associated with 1-year changes in physical activity and sedentary behaviours amongst this sample of British children. It appears that the provision of longer breaks, and more safety features on the route to school may help support maintenance of activity levels in children, as may having more same-age peers. The findings suggest how schools have an important role to play in preventing declines in children’s physical activity. Further work is needed to confirm our findings in other settings and investigate the counter-intuitive associations we observed.

## Competing interests

The authors declare they have no competing interests.

## Authors’ contributions

JM participated in the design of the study, lead on the analysis, and drafted the manuscript, APJ participated in the design of the study, supervised the analysis and drafted the manuscript, EVS and KK participated in the design of the study, provided the data, and contributed to the manuscript, NJ lead on the school audits and contributed to the manuscript, FH generated the GIS variables and contributed to the manuscript, SJ participated in the design of the study and contributed to the manuscript. All authors read and approved the final manuscript.
